# Lateral Habenula Glutamatergic Neurons Modulate Isoflurane Anesthesia in Mice

**DOI:** 10.3389/fnmol.2021.628996

**Published:** 2021-03-04

**Authors:** Chengxi Liu, Junxiao Liu, Liang Zhou, Haifeng He, Yu Zhang, Shuang Cai, Chengdong Yuan, Tianyuan Luo, Jijian Zheng, Tian Yu, Mazhong Zhang

**Affiliations:** ^1^Department of Anesthesiology, Shanghai Children’s Medical Center, Shanghai Jiao Tong University School of Medicine, Shanghai, China; ^2^Guizhou Key Laboratory of Anaesthesia and Organ Protection, Affiliated Hospital of Zunyi Medical University, Zunyi, China; ^3^Guizhou Key Laboratory of Brain Science, Zunyi Medical University, Zunyi, China; ^4^Department of Anesthesiology, Affiliated Hospital of Zunyi Medical University, Zunyi, China; ^5^Department of Anesthesiology, The Second Affiliated Hospital of Zunyi Medical University, Zunyi, China

**Keywords:** lateral habenula, isoflurane, glutamatergic, rostromedial tegmental, nucleus, induction time, recovery time

## Abstract

Since their introduction in the 1840s, one of the largest mysteries of modern anesthesia are how general anesthetics create the state of reversible loss of consciousness. Increasing researchers have shown that neural pathways that regulate endogenous sleep–wake systems are also involved in general anesthesia. Recently, the Lateral Habenula (LHb) was considered as a hot spot for both natural sleep–wake and propofol-induced sedation; however, the role of the LHb and related pathways in the isoflurane-induced unconsciousness has yet to be identified. Here, using real-time calcium fiber photometry recordings *in vivo*, we found that isoflurane reversibly increased the activity of LHb glutamatergic neurons. Then, we selectively ablated LHb glutamatergic neurons in Vglut2-cre mice, which caused a longer induction time and less recovery time along with a decrease in delta-band power in mice under isoflurane anesthesia. Furthermore, using a chemogenetic approach to specifically activate LHb glutamatergic neurons shortened the induction time and prolonged the recovery time in mice under isoflurane anesthesia with an increase in delta-band power. In contrast, chemogenetic inhibition of LHb glutamatergic neurons was very similar to the effects of selective lesions of LHb glutamatergic neurons. Finally, optogenetic activation of LHb glutamatergic neurons or the synaptic terminals of LHb glutamatergic neurons in the rostromedial tegmental nucleus (RMTg) produced a hypnosis-promoting effect in isoflurane anesthesia with an increase in slow wave activity. Our results suggest that LHb glutamatergic neurons and pathway are vital in modulating isoflurane anesthesia.

## Introduction

In the clinic, general anesthetics have been widely used since 1846. Nonetheless, the precise mechanisms by which the general anesthetics cause the sudden reversible loss of consciousness, remain to be pinpointed. The sedative effects of anesthetics such as drowsiness, calmness and reduction of motor tone are behaviorally similar to the features of the non-rapid eye movement (NREM) period of sleep (Franks and Zecharia, 2011; van der Meij et al., 2019). Some whole-brain imaging studies have also shown that the states of “unconsciousness” during deep sleep and anesthesia are remarkably similar (Franks, 2008). Moreover, similar slow-wave spatiotemporal properties during NREM sleep and isoflurane anesthesia suggest that both types of slow-waves are based on related processes (van der Meij et al., 2019). Recently, growing evidence proved that general anesthesia-induced unconsciousness and natural sleep shared some neural networks (Zhong et al., 2017; van der Meij et al., 2019; Zhang et al., 2019; Liu et al., 2020b).

The lateral Habenula (LHb), a component of the diencephalon, plays a central role in connecting the forebrain and midbrain and controlling both the dopaminergic system and the serotonergic system (Aizawa et al., 2012; Wagner et al., 2016; Hu et al., 2020). The LHb is mostly glutamatergic (Aizawa et al., 2012), predominantly expressing the mRNA of vesicular glutamate transporter 2 (Vglut 2) (Wagner et al., 2016). It mainly functions in reward processing, stress adaptation, sleep and circadian rhythm regulation (Mendoza, 2017; Gelegen et al., 2018; Flanigan et al., 2020; Hu et al., 2020). The LHb has dense reciprocal connections with arousal- and sleep-relevant structures, including the lateral hypothalamic area (LHA), lateral preoptic area (LPO), basal forebrain (BF), ventral tegmental area (VTA), and rostromedial tegmental nucleus (RMTg) (Mendoza, 2017; Hu et al., 2020). Lesion of the LHb induces a reduction in sleep rebound time after sleep deprivation (Zhang et al., 2016) and shortens the duration of hippocampal theta oscillations (Aizawa et al., 2013b). Novel research shows that lesions of LHb glutamatergic neurons heighten resistance to propofol-induced anesthesia, while selective activation of the LHb accelerates propofol anesthesia (Gelegen et al., 2018). These studies indicated that the LHb participated in sleep regulation and propofol anesthesia. However, it remains to be elucidated whether the LHb is causally involved in the process of unconsciousness induced by other anesthetic drugs.

In the present study, we characterized and investigated how LHb glutamatergic neurons regulate isoflurane anesthesia. Calcium fiber photometry recordings were used to examine the neural activities of LHb glutamatergic neurons in the process of isoflurane-induced anesthesia. Then, to clarify the function of LHb glutamatergic neurons in anesthesia, we selectively lesioned glutamatergic neurons. Subsequently, LHb glutamatergic neurons were chemogenetically stimulated to activate or inactivate during isoflurane anesthesia. Finally, we searched for the LHb downstream pathways mediating isoflurane anesthesia using optogenetic modulation. Our findings show that LHb glutamatergic neurons play a critical role in modulating isoflurane anesthesia.

## Materials and Methods

### Animals

This study was performed in accordance with the guidelines described in the Guide for the Care and Use of Laboratory Animals in China (No. 14924, 2001) and was approved by the Animal Care and Use Committees of Zunyi Medical University. Adult male and female Vglut2-IRES-Cre mouse littermates on a C57BL/6J background were used. C57BL/6J mice were provided by Changsha Tianqin Technology Co., Ltd. (Changsha, China). Mice were housed in standard chambers within an SPF laboratory animal room (12/12-hour light/dark cycle (light on at 6:00 am); 23 ± 2°C; relative humidity: 55% ± 2%). They were given free access to water and food (Jiangsu Xietong Pharmaceutical biology Co., Ltd., No. 1010009). To abate the confounding effects of circadian timing on the experimental results, all behavioral tests and electroencephalogram (EEG) experiments were performed between 18:00 and 24:00 p.m.

### Drugs

Isoflurane was purchased from RWD Life Science (Shenzhen, China). Pentobarbital and lidocaine were purchased from Chaohui Pharmaceutical (Shanghai, China). Clozapine *N*-oxide (CNO) was purchased from Sigma-Aldrich (United States, C0832). CY3 goat anti-rabbit IgG are products of Abcam Corp. (United States).

### Stereotaxic Surgery

Mice were anaesthetized with 1.4% isoflurane with oxygen (O_2_) at 1 L/min and then placed on a stereotaxic apparatus (RWD Life Science). Lidocaine (1%) was subcutaneously injected for local anesthesia before exposing the surface of the skull. Adeno-associated virus expressing Cre-inducible GcaMP (rAAV-hSyn-DIO-Gcamp6s), chemogenomic (AAV-EF1α-DIO-hM3Dq-eYFP and AAV-EF1α-DIO-hM4Di-eYFP), optogenetic (AAV-EF1α-DIO-ChR2-eYFP), eYFP (AAV-EF1α-DIO-eYFP), or DTA (AAV-CAG-DIO-DTA) (Brain-VTA, Wuhan, China) was injected into the LHb (150 nl/side; speed: 15 nl/min) (anterior-posterior [AP]: −1.40 mm, medial-lateral [ML]: ± 0.44 mm, and dorsal-ventral [DV]: −2.95 mm) according to the atlas of Paxinos and Franklin (2013) through a glass micropipette using a microsyringe pump and expressed for 21 days. Optic fibers were implanted unilaterally over the LHb/RMTg/VTA (VTA: anterior-posterior [AP]: −3.3 mm, medial-lateral [ML]: ±0.35 mm, dorsal-ventral [DV]: −4.25 mm; RMTg: anterior-posterior [AP]: −4.16 mm, medial-lateral [ML]: ±0.28 mm, dorsal-ventral [DV]: −4.3 mm),and secured with three skull screws and dental cement. Mice with verified viral infection sites and fiber placements in the LHb were included in the analysis.

### Calcium Fiber Photometry Recordings

Using a multichannel fiber photometry system (ThinkerTech Nanjing Bioscience Nanjing, China) equipped with a 480-nm excitation LED (3 W, CREE) and a dichroic mirror (DCC3420M; Thorlabs), the fluorescence signals of the GCaMP were recorded using multifunction data acquisition software (Thinker Tech Nanjing Bioscience Inc.). Simultaneously filtered at 40 Hz and digitalized at 500 Hz. An optical fiber (Newton Inc., China) integrated with an optical diverter (Doric Lenses) was used to transmit the light between the fiber photometry system and the implanted optical fiber (Luo et al., 2018, 2020; Liu et al., 2020a; Xu et al., 2020). One month later, 12 mice were subjected to record changes in GCaMP signals.

Before anesthesia, 100-second recording was completed. Next, the mice were anesthetized using 1.4% isoflurane; the moment of loss of righting reflex (LORR) and recovery of righting reflex (RORR) were marked, and the recording was stopped 10 min after RORR. Isoflurane anesthesia between administration and withdrawal of isoflurane was maintained for 25 min to ensure that the isoflurane concentration had equilibrated in the brain. Fiber photometry data were analyzed using MATLAB 2016a (MathWorks, Cambridge, United States). The values of fluorescence change (Δ*F*/*F*) were calculated using the following formula: (*F* − *F*_0_)/*F*_0_, where *F* is the test fluorescence signal and *F*_0_ is the basal signal (Luo et al., 2018, 2020; Liu et al., 2020a; Xu et al., 2020).

### Behavioral Tests

Loss of righting reflex and RORR time in mice is considered a standardized index of the general anesthesia induction and emergence times, respectively. Usually, the anesthesia induction time is regarded as the time to LORR in mice. For this reason, mice were placed into an anesthesia chamber (10 × 20 × 15 cm) that had been allowed to equilibrate for 10 min. Subsequently, the mice were induced and maintained by 1.4% isoflurane with 100% O_2_ at 1 l/min. An anesthesia monitor (Vamos; Drager Company, Germany) was connected to detect the concentration of isoflurane in the anesthesia chamber and an electric blanket with a rectal temperature probe was used to the bottom of the anesthesia chamber and was controlled at 37.5°C in the whole experiment. The mice were then removed from the chamber and allowed to emerge from anesthesia in an electric blanket. The period from the start of isoflurane treatment to LORR was deemed the LORR time, while the duration from the end of isoflurane infusion to RORR was defined as the RORR time.

For selective depletion of glutamatergic neurons in the LHb, Vglut2-IRES-Cre mice were bilaterally injected with AAV-CAG-DIO-DTA into the LHb area using an aseptic technique. For chemogenetic experiments, the mice in control, M3 and M4 group were injected either CNO (1 mg/ml, 1 mg/kg, i.p.) or saline (0.9%, equal volume, i.p.) 1 h before the behavioral test and EEG recording. There was at least 5-days rest between CNO and saline in the same mouse. For optogenetic experiments, we applied optical stimulation, which was performed by using a laser of 473 nm at 10 Hz for a duration of 10 ms, before the onset of induction and emergence in anesthesia (Figure 5A). The intensity of laser was tested with an optical power meter (PM100D, Thorlabs) and calibrated to 10 mW at the fiber tip. LORR, RORR, and EEG were recorded under isoflurane anesthesia. All mice were sacrificed and subjected to immunofluorescence to verify the virus expression and specific transfection after the experiments were performed.

**FIGURE 1:**
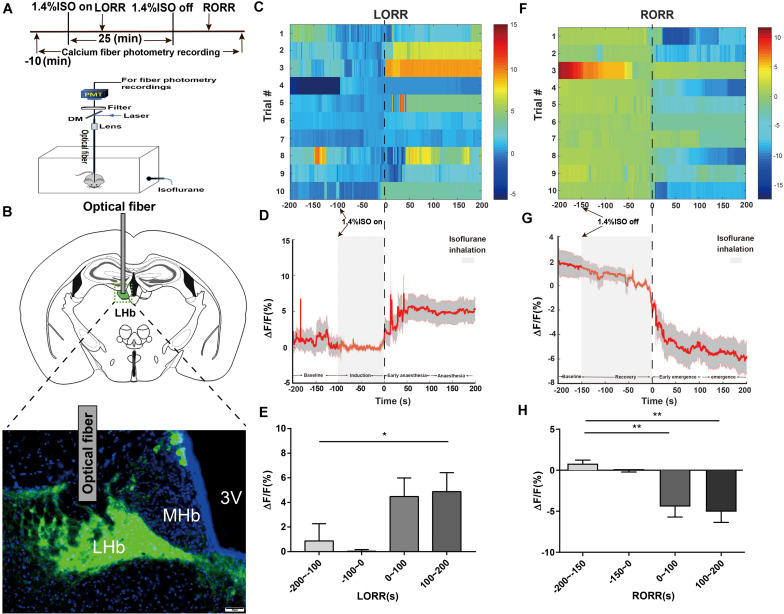
Phase-dependent calcium alterations in LHb glutamatergic neurons during isoflurane anesthesia. **(A)** Top: Timeline for quantifying the LORR and RORR with isoflurane. Bottom: Schematic diagram depicting fiber photometry recording during isoflurane anesthesia in freely moving mice. **(B)** Schematic of establishing calcium signal recording model into the LHb in Vglut2-Cre mice, and representative image of the LHb expressing GCaMP6s and optical fiber implanting sites (scale bar = 50 μm). **(C)** Fluorescence calcium signals aligned to isoflurane-induced loss of righting reflex [LORR were represented the moment of 0, each row plots one trial and a total of 10 trials are illustrated. Color scale at the right represents the value of Δ*F*/*F*(%)]. **(D)** Mean (red trace) ± SEM (gray shading) indicating the average calcium transients during isoflurane induced LORR (*n* = 10). **(E)** The fluorescence calcium signals increased after isoflurane-induced unconsciousness [The baseline (wake: –200 to –100 s) vs anesthesia period (100 to 200 s), *P* = 0.0475, *n* = 10, Dunnett’s multiple comparisons test after one-way ANOVA]. **(F)** Fluorescence calcium signals aligned to isoflurane-induced recovery of righting reflex [RORR were represented the moment of 0, each row plots one trial and a total of 10 trials are illustrated. Color scale at the right represents the value of ΔF/F (%)]. **(G)** Mean (red trace) ± SEM (gray shading) showing the transients of average calcium signals during isoflurane-induced RORR (*n* = 10). **(H)** The fluorescence calcium signals sharply decreased during the transition from isoflurane-induced anesthesia to arousal [The baseline (anesthesia: –200 to –150 s) vs early emergence period (0 to 100 s); *P* = 0.0055; The baseline: (anesthesia: –200 to –150s) vs emergence period (100 to 200 s), *P* = 0.0026; assessed by one-way ANOVA with Dunnett’s multiple comparisons test; *n* = 10, *P < 0.05, **P < 0.01].

**FIGURE 2:**
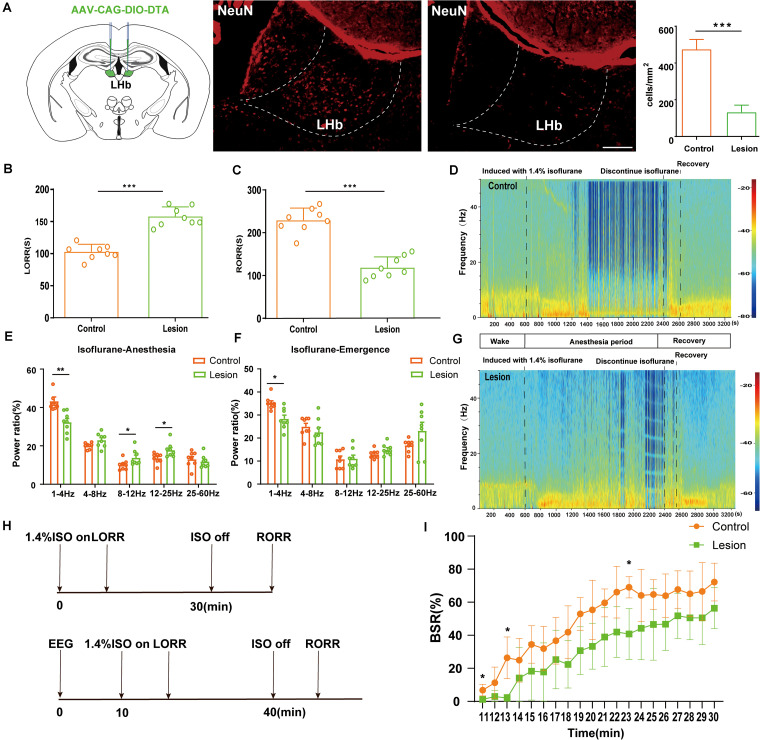
Bilateral lesion of LHb glutamatergic neurons on LORR and RORR time of isoflurane anesthesia. **(A)** Schematic representation of bilateral AAV injections into the LHb. Image showing NeuN (neuron-specific nuclear protein) staining from a mouse with specific LHb lesion using AAV-CAG-DIO-DTA (scale bar, 100 μm). The lesion group animals were selectively ablated LHb glutamatergic neurons. **(B)** Induction time and **(C)** recovery time in the lesion and sham groups (LORR: control group vs lesion group, *n* = 8; *P* = 0.000001 by independent-samples *t*-test; RORR: control group vs lesion group, *n* = 8; *P* = 0.00001 by independent-samples *t*-test *n* = 8 per group). **(D)** Spectrograms of EEG power during the isoflurane anesthesia period in the control group. **(E)** In the isoflurane anesthesia period, the power ratios of the δ band (1–4 Hz), α band (8–12 Hz) and β (12–25 Hz) in the lesion group were significantly changed (δ band: lesion group vs control group; *P* = 0.002 by independent-samples *t*-test; α band: lesion group vs control group; *P* = 0.045 by independent-samples *t*-test; β band: lesion group vs control group; *P* = 0.020 by independent-samples *t*-test, *n* = 8 per group). **(F)** Lesion of LHb glutamatergic neurons displaying a significant decrease in the δ band (1–4 Hz) between the two groups (lesion group vs control group; *P* = 0.03 by independent-samples *t*-test). **(G)** Spectrograms of EEG power during the isoflurane anaesthesia period in the lesion group. **(H)** Protocol for behavioral and electroencephalogram (EEG) recording of induction and emergence times. **(I)** BSR at 20 min before cessation of isoflurane in M3-NS or M3-CNO. BSR is plotted at each minute (*n* = 8), using two-way analysis of variance (ANOVA) followed by *post hoc* Bonferroni’s multiple comparisons: *F*(1, 14) = 15.06, *P* = 0.0017 (*n* = 8 per group; mean ± SD; *P < 0.05, **P < 0.01, and ***P < 0.001).

**FIGURE 3:**
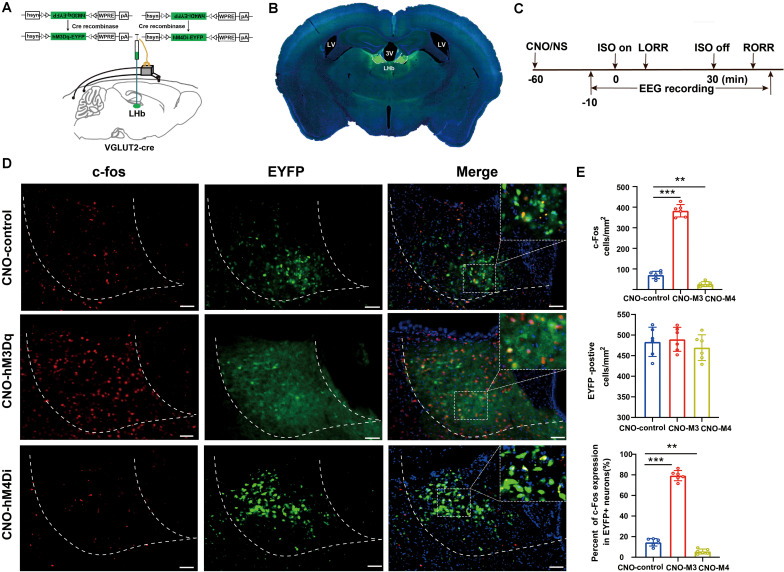
Activation/inactivation of LHb glutamatergic neurons induced LHb c-Fos expression during isoflurane anesthesia. **(A)** Surgical manipulations and experimental schematic for chemogenetic stimulation of LHb glutamatergic neurons. **(B)** Representative images of chemogenetic virus expression in LHb (scale bar, 1 mm). **(C)** Timelines of isoflurane anesthesia-related behavioral and EEG tests measuring induction time (LORR) and emergence time (RORR). **(D)** Representative images of c-Fos expression (red) and EYFP (green) in control (EYFP), hM3Dq-CNO and hM4Di-CNO mice groups after treatment with CNO (scale bar, 100 μm). **(E)** CNO administration decreased c-Fos expression in mCherry + neurons by 74%. Quantification of CNO administration induced the number of c-fos-positive neurons, the number of EYFP-positive neurons, and the percent of c-Fos expressing in EYFP-positive neurons after CNO administration (CNO administration significantly increased c-Fos expression in EYFP + neurons, *P* < 0.001 by Bonferroni’s *post hoc* test after one-way ANOVA; CNO administration decreased c-Fos expression in EYFP + neurons, *P* = 0.002 by Bonferroni’s *post hoc* test after one-way ANOVA; *n* = 6 per group; mean ± SD; **P < 0.01 and ***P < 0.001).

**FIGURE 4:**
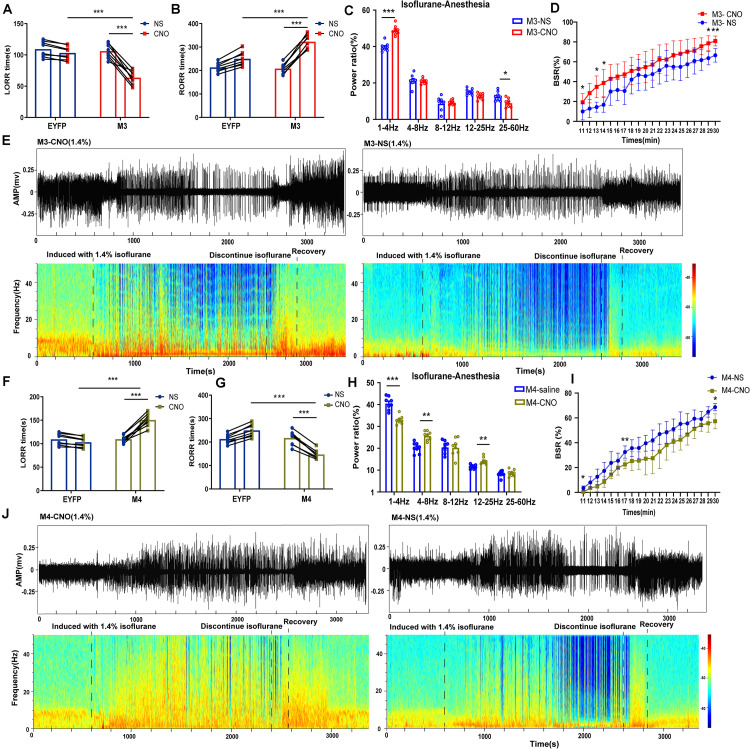
Chemogenetic manipulation of LHb glutamatergic neurons changed induction and arousal from isoflurane. **(A)** Chemogenetic activation of LHb glutamatergic neurons shortened loss of righting reflex (LORR) time from 1.4% isoflurane anesthesia. (M3-NS vs M3-CNO, *P* = 0.000046, paired *t*-test; Control-CNO vs. hM3Dq-CNO, *P* = 0.000003, independent-simples *t*-test; *n* = 8 per group). **(B)** Chemogenetic activation of LHb glutamatergic neurons prolonged the recovery of righting reflex (RORR) time from 1.4% isoflurane anesthesia (M3-NS vs M3-CNO, *P* = 0.000002, paired *t*-test; EYFP-CNO vs M3-CNO, *P* = 0.000253, independent-simples *t*-test; *n* = 8per group). **(C)** The power distribution of EEG frequency bands in M3-NS or M3-CNO group under 1.4% isoflurane anesthesia; (δ band: *P* = 0.000044, paired *t*-test; α band: *P* = 0.011, paired *t*-test). **(D)** BSR at 20 min before cessation of isoflurane in M3-NS or M3-CNO. BSR is plotted at each minute (*n* = 8), using two-way analysis of variance (ANOVA) followed by *post hoc* Bonferroni’s multiple comparisons: *F*(1, 14) = 8.406, *P* = 0.0117. **(E)** Representative EEG waveforms and spectrograms EEG power of M3-CNO and M3-NS under 1.4% isoflurane anesthesia. **(F)** Chemogenetic inactivation of LHb glutamatergic neurons prolonged the induction time from 1.4% isoflurane anesthesia (M4-NS vs M4-CNO, *P* = 0.000052, paired *t*-test; EYFP-CNO vs M4-CNO, *P* = 0.000002, independent-simples *t*-test). **(G)** Chemogenetic inactivation of LHb glutamatergic neurons shortened the recovery time from 1.4% isoflurane anesthesia (M4-NS vs M4-CNO, *P* = 0.000014, paired *t*-test; EYFP-CNO vs M4-CNO, *P* = 0.000003, independent-simples *t*-test). **(H)** The power distribution of EEG frequency bands during chemogenetic inactivation of LHb glutamatergic neurons under 1.4% isoflurane anesthesia (M4-NS vs M4-CNO: δ band, *P* = 0.000312, paired *t*-test; θ band, *P* = 0.01, paired *t*-test; β band, *P* = 0.01, paired *t*-test). **(I)** BSR at 20 min before cessation of isoflurane in M4-NS or M4-CNO. BSR is plotted at each minute (*n* = 8), using two-way analysis of variance (ANOVA) followed by *post hoc* Bonferroni’s multiple comparisons: *F*(1, 14) = 11.66, *P* = 0.0042. **(J)** Representative EEG waveforms and spectrograms EEG power of M4-CNO and M4-NS under 1.4% isoflurane anesthesia (*P < 0.05; **P < 0.01; ***P < 0.001; *n* = 8 per group).

**FIGURE 5:**
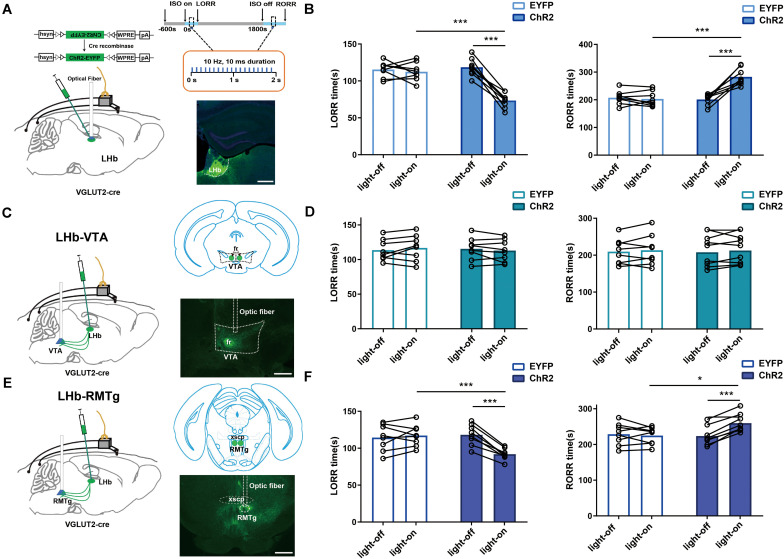
LHb glutamatergic neurons modulated isoflurane anesthesia through the LHb-RMTg pathway. **(A)** Left: Schematic of optogenetic stimulation of ChR2-expressing LHb glutamatergic neurons with EEG recordings; Right: Protocol for optogenetic activation during isoflurane anesthesia (Top). Image of ChR2-expressing LHb glutamatergic neurons (Bottom, scale bar, 400 μm). **(B)** Optical activation of LHb glutamatergic neurons shortened induction time (LORR: EYFP-light-on vs ChR2-light-on, *P* = 0.000008, independent-simples *t*-test; ChR2-light-on vs ChR2-light-off, *P* = 0.000043, paired *t*-test) and prolonged emergence time from 1.4% isoflurane anesthesia (RORR: EYFP-light-on vs ChR2-light-on, *P* = 0.000363, independent-simples *t*-test; ChR2-light-on vs ChR2-light-off, *P* = 0.000074, paired *t*-test). **(C)** Schematic of optogenetic stimulation of ChR2-expressing glutamatergic terminals in the ventral tegmental area (VTA) with EEG recordings (left); image of ChR2 expression in the VTA (right, scale bar, 400 μm). **(D)** Optical stimulation of glutamatergic terminals in the VTA had no impact on the induction and emergence time during isoflurane anesthesia. **(E)** Schematic of optogenetic stimulation of ChR2-expressing glutamatergic terminals in the rostromedial tegmental nucleus (RMTg) with EEG recordings (left); image of ChR2 expression in the RMTg (right, scale bar, 400 μm). **(F)** Optical activation of glutamatergic terminals in the RMTg accelerated the induction (LORR: EYFP-light-on vs ChR2-light-on, *P* = 0.000154, independent-simples *t*-test; ChR2-light-on vs ChR2-light-off, *P* = 0.000049, paired *t*-test) and slacked the emergence from 1.4% isoflurane anesthesia (RORR: EYFP-light-on vs ChR2-light-on, *P* = 0.0218, independent-simples *t*-test; ChR2-light-on vs ChR2-light-off, *P* = 0.0007, paired *t*-test; *P < 0.05; ***P < 0.001; *n* = 8 per group).

### EEG Recording and Spectral Analysis

Electroencephalograms were recorded at least 5 days after the behavioral test to allow recovery from anesthesia. The multichannel signal acquisition system (Appolo, Bio-Signal, Technologies, United States) was used to acquire EEG signals. The EEG signals were collected and filtered between 0.1 and 300 Hz. Before induction, the EEG signals were recorded for 10 min. Then, the EEG signals were continuously recorded from the 10 min before administration to recovery from isoflurane anesthesia, including anesthesia was maintained for 30 min. For lesion experiments, power spectrum analysis was conducted on data from the period of anesthesia maintenance (10 min before cessation of isoflurane) and the recovery period (10 min after cessation of isoflurane, Figure 2H). For chemogenetic experiments, we selected data from the period of anesthesia maintenance (10 min before cessation of isoflurane) for analysis (Figure 3C). Relative powers in the different frequency bands were computed by averaging the signal power across the frequency range of each band (δ: 1–4 Hz, θ: 4–8 Hz, α: 8–12 Hz, β: 12–25 Hz, and γ: 25–60 Hz) and then dividing by the total power from 1 to 60 Hz as previous studies (Luo et al., 2018; Liu et al., 2020a). Spectrogram was bandpass filtered at 0.1 to 50 Hz. Spectrograms were constructed using multitaper methods implemented using the Chronux toolbox in MATLAB 2016a (MathWorks, Cambridge, United States). An improved method for burst suppression rate (BSR, 20 min before cessation of isoflurane) was analysis in MATLAB 2016a (MathWorks, Cambridge, United States) as previous studies (Li et al., 2019; Wang et al., 2020).

### Histological Localization of the Cannula Position and Immunohistochemistry

Isoflurane (2%) was used to anesthetize the mice, and lidocaine (2%) was injected subcutaneously to induce local anesthesia. After deep anesthesia, the mice were transcranially infused with 300 ml of PBS, followed by 250 ml of 4% PFA in PBS. Their brains were removed and fixed in 4% PFA in PBS overnight at 4°C. The brains were later transferred to 30% sucrose in PBS at 4°C until they sank. The brains of the microinjection groups were coronally sectioned into 30-μm slices in a cryostat (CM1950; Leica, Germany) to validate the microinjection sites according to the mouse brain atlas (Paxinos and Franklin, 2013). For the lesion experiment, the brains were sectioned into slices, as described previously (Liu et al., 2020). The glutamatergic neurons of the LHb were stained immunohistochemically using an anti-NeuN antibody (MAB377, Millipore). The number of neuronal lesions in the LHb area was then calculated in a blinded manner by comparing positively immunostained neurons in Image J. NeuN-positive neurons were counted in a 0.5 × 0.5 mm box. Cell counting was performed on three adjacent sections (separated by 90 μm) of the brain, the average counting per section was used to represent the data.

For immunofluorescence, hM3Dq- and hM4Di-expressing mice (*n* = 8) were injected with CNO (1 mg/ml, 1 mg/kg, i.p.) or saline (0.9%, equal volume, i.p.) and then kept in their home cage for 2 h before perfusion. The brain sections were first incubated in blocking solution (PBS containing 2.5% normal goat serum, 1.5% bovine serum albumin and 0.1% Triton X-100) for 2 h at room temperature. Then the sections were incubated with the primary antibody (c-Fos staining, No. 226, Synaptic Systems) in blocking solution overnight at 4°C, and washed with PBST (PBS with 0.1% Triton X-100, vol/vol). Sections were then incubated with the secondary antibody (goat anti-rabbit Alexa 594 and Alexa 488, 1:1,000, Invitrogen) at room temperature for 2 h. After another wash with PBS, the sections were mounted on glass slides and cover-slipped with mounting media (Gold antifade reagent with DAPI, Life Technologies, United States). All images were captured by Olympus BX63 virtual microscopy system. The EYFP-positive neurons and c-Fos-positive cells were counted on alternate sections on both sides of the brain in the LHb area in a 0.5 × 0.5 mm box (approximately from bregma −1.06 to −196 mm, as per the mouse atlas of Paxinos and Franklin, 2013, *n* = 6, 1–2 sections per mouse).

### Data Analysis

All statistical analyses were performed by the GraphPad Prism software package, version 6.0 (GraphPad Software Inc., San Diego, CA, United States). All data were subject to tests for normality. The differences in cell count, LORR and RORR time were also detected using the independent-samples t-tests between the lesion and sham groups. Furthermore, paired Student’s *t*-tests were used to analyse differences in calcium signals between the pre- and post-events periods, as well as the change in LORR and RORR times for chemogenetic and optogenetic experiments within group (CNO vs NS or light-on vs light-off). Independent-samples *t*-test were applied in the analysis of c-Fos expression and neuron numbers between groups. Moreover, Independent-samples *t*-test were also used to the comparison of LORR times and RORR times in optogenetic or chemogenetic experiments between groups (EYFP-CNO vs M3/M4-CNO or EYFP-NS vs M3/M4-NS) in isoflurane anesthesia. For the changes of EEG power bands, two-way ANOVA followed by Bonferroni *post hoc* test was used to analyse BSR in the lesion or chemogenetic experiments. Data are presented as the mean ± SD or mean ± SEM. In all cases, *P*-values < 0.05 were considered significant.

## Results

### Population Activities of LHb Glutamatergic Neurons Increased in the Isoflurane Anesthesia

To investigate the real-time activity of the LHb glutamatergic neurons during isoflurane anesthesia, we injected Cre-dependent AAV-hSyn-DIO-Gcamp6s into LHb neurons of Vglut2-IRES-Cre mice (Figure 1B) and used fiber photometry to record changes in Ca^2+^ signals *in vivo* during isoflurane anesthesia (Figure 1A).

During isoflurane anesthesia induction, we analyzed calcium signals in four sections: baseline (wake: −200 to −100 s), induction period (−100 to 0 s), early anesthesia period (0 to 100 s) and anesthesia period (100 to 200 s). As shown in Figures 1C–E, the Ca^2+^ signals exhibited almost no change during the induction period. The average Ca^2+^ signals of LHb glutamatergic neurons showed an active tendency during the early anesthesia period but not statistical significance compared to the baseline. During anesthesia-maintenance period, the average Ca^2+^ signals of LHb were highly active, suggesting that LHb glutamatergic neurons were activated during isoflurane anesthesia (Figure 1E). Four sections were analyzed during the recovery process, including baseline (anesthesia: −200 to −150 s), recovery period (−150 to 0 s), early emergence period (0 to 100 s) and emergence period (100 to 200 s). In the recovery process, the Ca^2+^ signals of LHb glutamatergic neurons began to decrease related to the moment of RORR (Figures 1F–H). Generally, our results indicate that LHb glutamatergic neurons are activated after LORR and inhibited during the emergence process, hinting that isoflurane activated the activity of LHb in state-dependent manner.

### Lesion of LHb Glutamatergic Neurons in Isoflurane Anesthesia Slowed Down Isoflurane-Induced Anesthesia

To explore the role of the LHb in isoflurane anesthesia, we injected AAV-CAG-DIO-DTA virus vector into Vglut2-Cre mice to selectively ablate LHb glutamatergic neurons. As shown in Figure 2A, after 4 weeks of virus injection, the neuron number in the LHb was lower after lesion treatment than in the normal group. For lesion group, a longer LORR time (Figure 2B) and a shorter RORR time were found (Figure 2C) compared with that in control group. Meanwhile, cortical EEG of the sham and lesion groups were recorded during isoflurane anesthesia (Figure 2H). During isoflurane anesthesia, the power ratio of the δ wave was notably lower in the lesion group than in the sham group, whereas the power ratio of the α and β waves were higher in the lesion group than in the sham group (Figures 2D,E). During the recovery period, the δ wave of the lesion group significantly decreased compared to that of sham group (Figures 2F,G), while the other bands were not significantly different. Additionally, lesion of the LHb glutamatergic neurons reduces BSR during isoflurane anesthesia (Figure 2I). These results suggest that LHb lesion reduced the hypnotic effect of isoflurane anesthesia.

### Chemogenetic Activation of Glutamatergic LHb Neurons Promoted Isoflurane-Induced Anesthesia

To specifically activate glutamatergic neurons, we injected AAV-Ef1α-DIO-hM3Dq-EYFP and AAV-Ef1α-DIO-EYFP vectors into the LHb of Vglut2-Cre mice (Figure 3A). Immunofluorescence images validated the virus transfection in LHb glutamatergic neurons (Figure 3B). CNO pretreatment significantly promoted c-fos expression in LHb glutamatergic neurons (Figures 3D,E). During isoflurane anesthesia, chemogenetic activation of LHb glutamatergic neurons significantly reduced the induction time between the hM3Dq-CNO group and the hM3Dq-saline group, as well as between the hM3Dq-CNO group and the EGFP-CNO group (Figure 4A). A longer time to recovery was also found between the hM3Dq-CNO group and the hM3Dq-saline group, as well as between the hM3Dq-CNO group and the EGFP-CNO group (Figure 4B). EEG recordings were employed to further assess how LHb glutamatergic neurons affect the processes of isoflurane anesthesia. The simultaneous cortical EEG also altered in LHb glutamatergic neurons activated group (Figure 4J). In hM3Dq-expressing animals, chemogenetic activation of LHb glutamatergic neurons induced the augment of total power percentages of the δ wave (1–4 Hz) and the reduction of total power percentages of the γ wave (25–60 Hz) (Figure 4C), relative to the hM3Dq-saline group. Averaged BSR increased during the last 20 min of chemogenetic activation, compared with the hM3Dq-saline group (Figure 4D). These results indicated that activation of LHb glutamatergic neurons promotes isoflurane anesthesia.

### Chemogenetic Inactivation of Glutamatergic LHb Neurons Increased Tolerance to Isoflurane

We next inactivated LHb glutamatergic neurons by injecting AAV-Ef1α-DIO-hM4Di-EYFP and AAV-Ef1α-DIO-EYFP virus into Vglut2-Cre mice (Figure 3A). Functional expression of hM4Di mice was verified by staining for c-fos expression in LHb glutamatergic neurons. We found that c-fos expression was decreased in the LHb after CNO injection in hM4Di mice (Figures 3D,E). Chemogenetic inactivation of LHb glutamatergic neurons induced a longer duration to produce general anesthesia between the hM4Di -CNO group and the hM4Di-saline group, as well as between the hM4Di-CNO group and the control-CNO group (Figure 4F), and led to a faster emergence from isoflurane anesthesia (Figure 4G). Chemogenetic inhibition of LHb glutamatergic neurons reduced the total power percentages of δ band (1–4 Hz) and augmented the power ratio in θ band (4–8 Hz) and β band (12–25 Hz) during isoflurane anesthesia, without affecting another band (Figure 4H). As shown in Figure 4I, BSR during deep anesthesia (the last 20 min before cessation of isoflurane) was decreased by chemogenetic inactivation of LHb glutamatergic neurons compared with hM4Di-saline group. Consequently, chemogenetic inactivation of glutamatergic LHb neurons showed an increasing tolerance to isoflurane.

### Optogenetic Activation of Glutamatergic LHb Neurons or Glutamatergic LHb Neuron Axons in the RMTg Promotes Isoflurane Induced-Anesthesia

**FIGURE 6:**
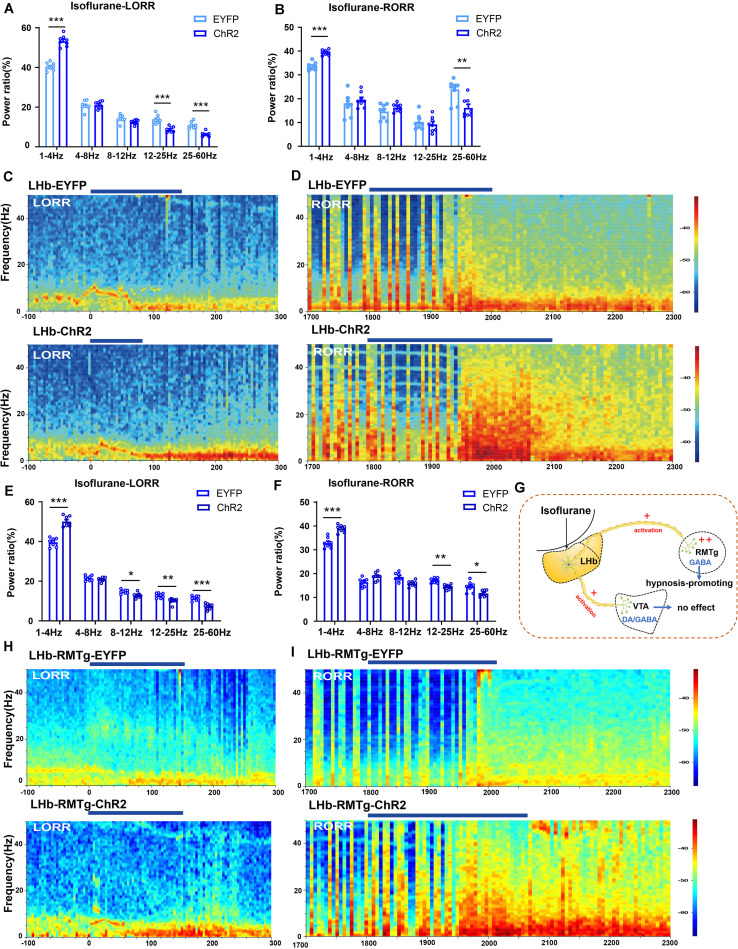
Optical activation of LHb glutamatergic neurons or the LHb-RMTg pathway affected the cortical EEG. **(A)** Optical activation of LHb glutamatergic neurons changed the cortical EEG during induction (EYFP-light-on vs ChR2-light-on, δ band: *P* = 0.0002, independent-simples *t*-test; β band: *P* = 0.00043, independent-simples *t*-test; γ band: *P* = 0.000161, independent-simples *t*-test). **(B)** Power percentage changes in cortical EEG during arousal from isoflurane with (EYFP-light-on vs ChR2-light-on, δ band: *P* = 0.000121, independent-simples *t*-test; γ band: *P* = 0.006, independent-simples *t*-test). **(C,D)** Representative EEG waveforms and spectrograms EEG power of LHb-EYFP and LHb-ChR2 group under 1.4% isoflurane anesthesia during isoflurane-induced process **(C)** and recovery process **(D)**. **(E)** Optogenetic activation of the LHb-RMTg pathway altered the power distribution of EEG frequency bands during the isoflurane-induced process (EYFP-light-on vs ChR2-light-on, δ band: *P* = 0.00002, independent-simples *t*-test; α band: *P* = 0.05, independent-simples *t*-test; β band: *P* = 0.001, independent-simples *t*-test; γ band: *P* = 0.000022, independent-simples *t*-test). **(F)** Optogenetic activation of the LHb-RMTg pathway altered the power distribution of EEG frequency bands during recovery process (EYFP-light-on vs ChR2-light-on, δ band: *P* = 0.0000037, independent-simples *t*-test; β band: *P* = 0.0001, independent-simples *t*-test; γ band: *P* = 0.0183, independent-simples *t*-test). **(G)** One mechanism for LHb modulate isoflurane anesthesia in mice through RMTg neurons. **(H,I)** Representative EEG waveforms and spectrograms EEG power in the LHb-RMTg-EYFP and LHb-RMTg-ChR2 during isoflurane-induced process **(H)** and recovery process **(I)** (*P < 0.05; **P < 0.01; ***P < 0.001; *n* = 8 per group).

We also used optogenetic methods to examine the causal role of the LHb, finding that optical activation of LHb glutamatergic neurons (Figure 6C) at the onset of induction and emergence in anesthesia prominently accelerated the induction process (Figure 5B) with an increase in total power percentages of δ waves and a decrease in total power percentages of β and γ waves (Figures 6A,D), and delayed the recovery time with a complementary increase of total power percentages of δ waves and the decrease in total power percentages of γ waves relative to the EYFP control (Figures 6B,E). Moreover, ChR2-EYFP expression indicated that LHb glutamatergic neurons send excitatory projections to multiple midbrain regions, including the VTA and RMTg (Figures 5C,E, Supplementary Figure 1), which have been reported to participate in sleep–wakefulness control.

To further identify the anatomic interaction between the LHb glutamatergic neurons and their downstream targets of projection, we separately performed rAAV-retro-hSyn-CRE-mCherry-WPRE-hGH retrograde tracing in the RMTg and VTA and found that the RMTg and VTA received direct inputs from the LHb (Supplementary Figure 2). Based on these results, we concluded that the LHb sends dense projections to the VTA and RMTg. Then, to test the functional role of this projection in isoflurane-induced anesthesia, we injected AAV-EF1α-DIO-ChR2-eYFP into the LHb of Vglut2-cre mice and implanted an optic fiber into the VTA and RMTg. Optical stimulation of LHb glutamatergic axonal terminals in the VTA had no obvious effects on induction and emergence during isoflurane anesthesia (Figure 5D). However, optical stimulation of the LHb-RMTg projections caused an acceleration from wake to isoflurane-induced unconsciousness (Figures 5E,F), accompanied by an augmentation of the total power percentages of δ waves and a decrease in the total power percentages of α-, β- and γ-waves (Figures 6E,H), and a slower recovery from isoflurane anesthesia (Figures 5E,F) with the augment of total power percentages of δ waves and the decrease of total power percentages of β and γ waves (Figures 6F,I), which is similar to the effects of optical activation of LHb glutamatergic neurons. These findings suggest that selective activation of LHb glutamatergic neurons or LHb to RMTg glutamatergic neurons is important to isoflurane anesthesia (Figure 6G).

## Discussion

In this study, we manipulated LHb glutamatergic neurons to elucidate the regulatory role of LHb in isoflurane anesthesia using calcium fiber photometry recordings, specific lesions, chemogenetics and optogenetics. The results revealed that neural activity in LHb glutamatergic neurons is positively correlated with isoflurane-anesthetized states. Specific lesions of LHb glutamatergic neurons led to a slower induction and promoted cortical arousal during isoflurane anesthesia. Similar results were observed after chemogenetic inhibition of LHb glutamatergic neurons. Compared to control mice, chemogenetic activation of LHb glutamatergic neurons resulted in a shorter induction time, and longer emergence time associated with isoflurane anesthesia. Moreover, using an optogenetic approach, instantaneous activation of LHb glutamatergic neurons or LHb glutamatergic axonal terminals in the RMTg but not the VTA produced both behavioral and simultaneous EEG changes under isoflurane anesthesia. Our results demonstrate that LHb glutamatergic transmission is sufficient to modulate the anesthetic state of isoflurane.

Calcium fiber photometry recording, one of the most sensitive and fastest methods to detect neuronal activities, revealed that LHb glutamatergic neurons were activated in isoflurane anesthesia and inhibited in the transition from anesthesia to arousal, implying that isoflurane-induced anesthesia may require excitation of the LHb. This finding is in agreement with a recent study in propofol which sedative doses of propofol – induced an increase of c-Fos expression in the LHb, although propofol had no impact on the resting membrane potential of LHb neurons in acute slices (Gelegen et al., 2018). Both *in vivo* and *in vitro* electrophysiological experiments demonstrated that the extracellular activity of LHb neurons had a higher firing rate during the day than at night (Zhao and Rusak, 2005; Sakhi et al., 2014). Consistent with the electrophysiological experiments, c-Fos protein expression displays a day-night activity difference in various rodent species such as hamsters, mice and rats (Tavakoli-Nezhad and Schwartz, 2006). A previous study also found that the c-Fos expression of stressful stimuli during sleep was an evident enhancement in the LHb compared to wake in rats (Chastrette et al., 1991). Moreover, specific ablation of the LHb resulted in a decrease in the duration in the rapid eye movement (REM) sleep phase (Aizawa et al., 2013b). The above studies show that the LHb has a modulatory impact on the sleep–wake cycle to some extent. Owing to its role in sleep–wake regulation, as well as isoflurane and propofol-induced activation of LHb neurons, LHb is proposed to be a convenient node on modulating general anesthesia.

To explore the contribution of LHb glutamatergic neurons in isoflurane anesthesia, we further manipulated LHb glutamatergic neurons by using chemogenetics and optogenetics. Interestingly, our results found that selective ablation or inhibition of LHb glutamatergic neurons caused a delay of induction accompanying by the reduction of slow wave activity and an acceleration of reanimation with cortical arousal in isoflurane anesthesia; On the contrary, chemogenetic or optogenetic activation of LHb glutamatergic neurons accelerated isoflurane anesthesia induction and delayed arousal, implying an effective role of the LHb in the promotion of isoflurane anesthesia. It is well accepted that glutamatergic neurons are the main excitatory neurons in the brain. Previous studies have shown that activation of glutamatergic neurons in LPO, BF or paraventricular nucleus (PVN) also induced an increase in wakefulness (Xu et al., 2015; Liu et al., 2020; Vanini et al., 2020). Moreover, Wang and our previous study found that activation of parabrachial nucleus (PB) glutamatergic neurons not only promotes continuous wakefulness the natural sleep wake cycles but also accelerated emergence from anesthesia (Luo et al., 2018). Unlike previous studies on glutamatergic neurons involved in arousal promotion, some studies on the contribution of LHb presented a hypnosis-promoting effect in general anesthesia and sleep (Aizawa et al., 2013b; Zhang et al., 2016; Mendoza, 2017; Gelegen et al., 2018). It has been widely reported that patients with depression with hyperactivation of the habenula presented shorter latency to the onset of REM sleep, longer duration of REM sleep and enhanced eye movement frequency during REM sleep (Aizawa et al., 2013a; Mendoza, 2017; Gold and Kadriu, 2019). Previous studies have suggested that lesions of the habenular output, fascicular retroflexus (fr) fibers, in rats decreased the amount of time spent in REM sleep (Valjakka et al., 1998). In sleep deprived rats, LHb lesions increased wake time and decreased NREM sleep time compared to before LHb lesion (Zhang et al., 2016). Additionally, several studies have shown that melatonin, a naturally occurring circadian hormone, mediated control of glutamatergic inputs to the LHb playing a key role in the modulation of various behaviors (Evely et al., 2016). It is reasonable to conceive the idea that LHb participates in the regulation of the normal sleep–wake cycle. Recently, Gelegen et al. (2018) specifically blocked output from the LHb induced an obvious fragmentation of NREM sleep and greatly lessened the sedative effects of propofol. Based on the above studies and our results, we speculate that LHb plays a vital role in the control of the sleep–wake cycle and general anesthesia, providing the correlative evidence for a mechanistic overlap of sleep and anesthesia.

How does the excitatory glutamatergic neurons in LHb play the hypnosis-promoting effect? As one of the major targets of LHb – VTA and GABAergic RMTg in our study (Supplementary Figure 2) – is intensively studied for its regulation of the sleep–wake cycle and general anesthesia. Plenty researches showed that LHb send a major projection to the GABAergic RMTg, a modulator of midbrain dopamine systems (Jhou et al., 2009; Stamatakis and Stuber, 2012; Stephenson-Jones et al., 2012; Brown et al., 2017; Mendoza, 2017; Hu et al., 2020). Studies also showed that negative signals in the LHb are inverted and transmitted by the GABAergic RMTg relay nucleus or local interneurons within the VTA and SNc, regulating positive and negative reward-based decision and motor activity (Matsumoto and Hikosaka, 2007; Huff and LaLumiere, 2015; Hu et al., 2020). So, we speculated that the excitatory input from the lateral habenula may play an important role in determining the activity of VTA dopamine neurons through GABAergic RMTg in isoflurane anesthesia.

Studies have shown that VTA dopaminergic neurons are involved in the sleep–wake transition and emergence from isoflurane anesthesia (Taylor et al., 2013; Oishi and Lazarus, 2017; Yang et al., 2018). Several remarkable observations in this found that optogenetic, chemogenetic or electrical stimulation dopamine neurons in the VTA could induce wakefulness from either natural sleep (Oishi and Lazarus, 2017) or isoflurane anesthesia (Taylor et al., 2013). In our study, optical stimulation of LHb or LHb glutamatergic terminals in the RMTg but not the VTA significantly decreased the induction time with the augmentation of delta wave. Huang and colleagues found that pharmacogenetic activation the RMTg increased NREM sleep time with an augment of slow-wave activity, and optical stimulation of the RMTg neurons’ terminals in VTA caused direct inhibitory regulation of VTA dopaminergic neurons using a whole-cell patch clamp approach (Yang et al., 2018). Many researches also showed that excitatory glutamatergic outputs from the LHb predominately inhibit VTA dopamine neuronal firing through an RMTg inhibitory mechanism (Jhou et al., 2009; Stamatakis and Stuber, 2012; Stephenson-Jones et al., 2012; Brown et al., 2017; Mendoza, 2017; Hu et al., 2020). In our study, the effects of direct activation of LHb on isoflurane anesthesia were mimicked by modulation of the LHb-RMTg pathway using an optogenetic approach. Considering the regulatory role of LHb in the dopaminergic system, we inferred that the hypnosis-promoting effect of LHb glutamatergic neurons is partly mediated by their projection to the RMTg, where the excited GABAergic neurons restrain the arousal effect of VTA dopamine neurons. Our results delineate a novel pathway for general anesthesia.

Although abundant excitatory glutamatergic projections from the LHb to the RMTg exist (Jhou et al., 2009; Proulx et al., 2018), an evident limitation of our study is that we did not use electrophysiological technology to directly assess the axonal effects of LHb neurons on the activity of the RMTg. In addition, we should further observe the role of RMTg in general anesthesia using Vgat-Cre (vesicular GABA transporter) mice to specifically target GABAergic neurons to expand on our results.

Collectively, our data provide experimental evidence that supports LHb glutamatergic neurons playing as a critical node in modulating isoflurane anesthesia via the LHb-RMTg glutamatergic pathway.

## Data Availability Statement

The raw data supporting the conclusions of this article will be made available by the authors, without undue reservation.

## Ethics Statement

The animal study was reviewed and approved by Zunyi Medical University, China [grant number: 2019(2)-289].

## Author Contributions

CL and JL completed data analysis and writing the manuscript. MZ, TY, and JZ were responsible for design. LZ and HH was responsible for calcium fiber photometry. YZ, SC, CY, and TL performed the behavioral tests and the electrophysiology recordings. All authors read and approved the final manuscript.

## Conflict of Interest

The authors declare that the research was conducted in the absence of any commercial or financial relationships that could be construed as a potential conflict of interest.

## Supplementary Material

Supplementary Figure 1 Projections resulting from injections of AAV-EF1α-DIO-eYFP into the LHb. **(A)** Representative images of LHb projection in VTA (scale bar = 100 μm). **(B)** Higher magnification photograph of **(A)** (scale bar = 100 μm). **(C)** Representative images of LHb projection in RMTg (scale bar = 100 μm). **(D)** Higher magnification photograph of **(C)** (scale bar = 100 μm). **(E)** Representative images of LHb projection in LDTg (scale bar = 100 μm). **(F)** Higher magnification photograph of **(E)** (scale bar = 100 μm).

Supplementary Figure 2 **(A)** Representative injection of rAAV-retro-hSyn-CRE-mCherry-WPRE-hGH in RMTg. **(B)** A representative image of the LHb-projecting RMTg neurons. **(C)** Representative injection of rAAV-retro-hSyn-CRE-mCherry-WPRE-hGH in VTA. **(D)** A representative image of the LHb-projecting VTA neurons (scale bars = 400 μm).

Supplementary Figure 3 Injection sites were localized to LHb. Virus-transfection sites from representative photomicrograph of a virus-stained coronal section throughout the rostral-caudal axis **(A)** LHb are represented by green on coronal schematics modified from the atlas of Franklin and Paxinos between bregma −1.06 mm and bregma −1.94 mm in mouse (Paxinos and Franklin, 2013). **(B)** Corresponding schematic of the virus transfections sites in LHb.

## References

[B1] Aizawa H. Cui W. Tanaka K. Okamoto H. (2013a). Hyperactivation of the habenula as a link between depression and sleep disturbance. *Front. Hum. Neurosci.* 7:826. 10.3389/fnhum.2013.00826 24339810 PMC3857532

[B2] Aizawa H. Kobayashi M. Tanaka S. Fukai T. Okamoto H. (2012). Molecular characterization of the subnuclei in rat habenula. *J. Comp. Neurol.* 520 4051–4066. 10.1002/cne.23167 22700183

[B3] Aizawa H. Yanagihara S. Kobayashi M. Niisato K. Takekawa T. Harukuni R. (2013b). The synchronous activity of lateral habenular neurons is essential for regulating hippocampal theta oscillation. *J. Neurosci.* 33 8909–8921. 10.1523/jneurosci.4369-12.2013 23678132 PMC6618841

[B4] Brown P. L. Palacorolla H. Brady D. Riegger K. Elmer G. I. Shepard P. D. (2017). Habenula-Induced inhibition of midbrain dopamine neurons is diminished by lesions of the rostromedial tegmental nucleus. *J. Neurosci.* 37 217–225. 10.1523/jneurosci.1353-16.2017 28053043 PMC5214632

[B5] Chastrette N. Pfaff D. W. Gibbs R. B. (1991). Effects of daytime and nighttime stress on Fos-like immunoreactivity in the paraventricular nucleus of the hypothalamus, the habenula, and the posterior paraventricular nucleus of the thalamus. *Brain Res.* 563 339–344. 10.1016/0006-8993(91)91559-j 1786549

[B6] Evely K. M. Hudson R. L. Dubocovich M. L. Haj-Dahmane S. (2016). Melatonin receptor activation increases glutamatergic synaptic transmission in the rat medial lateral habenula. *Synapse* 70 181–186. 10.1002/syn.21892 26799638

[B7] Flanigan M. E. Aleyasin H. Li L. Burnett C. J. Chan K. L. LeClair K. B. (2020). Orexin signaling in GABAergic lateral habenula neurons modulates aggressive behavior in male mice. *Nat. Neurosci.* 23 638–650. 10.1038/s41593-020-0617-7 32284606 PMC7195257

[B8] Franks N. P. (2008). General anaesthesia: from molecular targets to neuronal pathways of sleep and arousal. *Nat. Rev. Neurosci.* 9 370–386. 10.1038/nrn2372 18425091

[B9] Franks N. P. Zecharia A. Y. (2011). Sleep and general anesthesia. *Can. J. Anaesth.* 58 139–148. 21170623 10.1007/s12630-010-9420-3

[B10] Gelegen C. Miracca G. Ran M. Z. Harding E. C. Ye Z. Yu X. (2018). Excitatory pathways from the lateral habenula enable propofol-induced sedation. *Curr Biol* 28 580–587.e585. 29398217 10.1016/j.cub.2017.12.050 PMC5835141

[B11] Gold P. W. Kadriu B. (2019). A major role for the lateral habenula in depressive illness: physiologic and molecular mechanisms. *Front Psychiatry* 10:320. 10.3389/fpsyt.2019.00320 31231247 PMC6558383

[B12] Huff M. L. LaLumiere R. T. (2015). The rostromedial tegmental nucleus modulates behavioral inhibition following cocaine self-administration in rats. *Neuropsychopharmacol* 40 861–873. 10.1038/npp.2014.260 25257212 PMC4330500

[B13] Hu H. Cui Y. Yang Y. (2020). Circuits and functions of the lateral habenula in health and in disease. *Nat. Rev. Neurosci.* 21 277–295. 10.1038/s41583-020-0292-4 32269316

[B14] Jhou T. C. Fields H. L. Baxter M. G. Saper C. B. Holland P. C. (2009). The rostromedial tegmental nucleus (RMTg), a GABAergic afferent to midbrain dopamine neurons, encodes aversive stimuli and inhibits motor responses. *Neuron* 61 786–800. 10.1016/j.neuron.2009.02.001 19285474 PMC2841475

[B15] Li J. Li H. Wang D. Guo Y. Zhang X. Ran M. (2019). Orexin activated emergence from isoflurane anaesthesia involves excitation of ventral tegmental area dopaminergic neurones in rats. *Br. J. Anaesth.* 123 497–505. 10.1016/j.bja.2019.07.005 31399212

[B16] Liu C. Shi F. Fu B. Luo T. Zhang L. Zhang Y. (2020a). GABA(A) receptors in the basal forebrain mediates emergence from propofol anaesthesia in rats. *Int. J. Neurosci.* 11 1–13. 10.1080/00207454.2020.1840375 33174773

[B17] Liu C. Zhou X. Zhu Q. Fu B. Cao S. Zhang Y. (2020b). Dopamine neurons in the ventral periaqueductal gray modulate isoflurane anesthesia in rats. *CNS Neurosci. Ther.* 26 1121–1133. 10.1111/cns.13447 32881314 PMC7564192

[B18] Liu Y. Li Y. Yang B. Yu M. Zhang X. Bi L. (2020). Glutamatergic neurons of the paraventricular nucleus are critical for the control of wakefulness. *Neuroscience* 446 137–144. 10.1016/j.neuroscience.2020.08.024 32860935

[B19] Luo T. Cai S. Qin Z.-X. Yang S. C. Shu Y. Liu C. X. (2020). Basal forebrain cholinergic activity modulates isoflurane and propofol Anesthesia. *Front. Neurosci.* 14:1086. 10.3389/fnins.2020.559077 33192246 PMC7652994

[B20] Luo T. Yu S. Cai S. Zhang Y. Jiao Y. Yu T. (2018). Parabrachial neurons promote behavior and electroencephalographic arousal from general anesthesia. *Front. Mol. Neurosci.* 11:420. 10.3389/fnmol.2018.00420 30564094 PMC6288364

[B21] Matsumoto M. Hikosaka O. (2007). Lateral habenula as a source of negative reward signals in dopamine neurons. *Nature* 447 1111–1115. 17522629 10.1038/nature05860

[B22] Mendoza J. (2017). Circadian neurons in the lateral habenula: clocking motivated behaviors. *Pharmacol. Biochem. Behav.* 162 55–61. 10.1016/j.pbb.2017.06.013 28666896

[B23] Oishi Y. Lazarus M. (2017). The control of sleep and wakefulness by mesolimbic dopamine systems. *Neurosci. Res.* 118 66–73. 10.1016/j.neures.2017.04.008 28434991

[B24] Paxinos G. Franklin K. B. J. (2013). *Paxinos and Franklin’s the Mouse Brain in Stereotaxic Coordinates.* Amsterdam: Elsevier/AP.

[B25] Proulx C. D. Aronson S. Milivojevic D. Molina C. Loi A. Monk B. (2018). A neural pathway controlling motivation to exert effort. *Proc. Natl. Acad. Sci. U.S.A.* 115 5792–5797. 10.1073/pnas.1801837115 29752382 PMC5984527

[B26] Sakhi K. Wegner S. Belle M. D. Howarth M. Delagrange P. Brown T. M. (2014). Intrinsic and extrinsic cues regulate the daily profile of mouse lateral habenula neuronal activity. *J. Physiol.* 592 5025–5045. 10.1113/jphysiol.2014.280065 25194046 PMC4259541

[B27] Stamatakis A. M. Stuber G. D. (2012). Activation of lateral habenula inputs to the ventral midbrain promotes behavioral avoidance. *Nat. Neurosci.* 15 1105–1107. 10.1038/nn.3145 22729176 PMC3411914

[B28] Stephenson-Jones M. Floros O. Robertson B. Grillner S. (2012). Evolutionary conservation of the habenular nuclei and their circuitry controlling the dopamine and 5-hydroxytryptophan (5-HT) systems. *Proc. Natl. Acad. Sci. U.S.A.* 109 E164–E173. 22203996 10.1073/pnas.1119348109 PMC3271889

[B29] Tavakoli-Nezhad M. Schwartz W. J. (2006). Hamsters running on time: is the lateral habenula a part of the clock? *Chronobiol. Int.* 23 217–224. 10.1080/07420520500521947 16687295

[B30] Taylor N. E. Chemali J. J. Brown E. N. Solt K. (2013). Activation of D1 dopamine receptors induces emergence from isoflurane general anesthesia. *Anesthesiology* 118 30–39. 10.1097/aln.0b013e318278c896 23221866 PMC3527840

[B31] Valjakka A. Vartiainen J. Tuomisto L. Tuomisto J. T. Olkkonen H. Airaksinen M. M. (1998). The fasciculus retroflexus controls the integrity of REM sleep by supporting the generation of hippocampal theta rhythm and rapid eye movements in rats. *Brain Res. Bull.* 47 171–184. 10.1016/s0361-9230(98)00006-9 9820735

[B32] van der Meij J. Martinez-Gonzalez D. Beckers G. J. L. Rattenborg N. C. (2019). Neurophysiology of avian sleep: comparing natural sleep and isoflurane anesthesia. *Front. Neurosci.* 13:262. 10.3389/fnins.2019.00262 30983954 PMC6447711

[B33] Vanini G. Bassana M. Mast M. Mondino A. Cerda I. Phyle M. (2020). Activation of preoptic GABAergic or glutamatergic neurons modulates sleep-wake architecture, but not anesthetic state transitions. *Curr. Biol.* 30 779–787.e774. 32084397 10.1016/j.cub.2019.12.063 PMC7156032

[B34] Wagner F. French L. Veh R. W. (2016). Transcriptomic-anatomic analysis of the mouse habenula uncovers a high molecular heterogeneity among neurons in the lateral complex, while gene expression in the medial complex largely obeys subnuclear boundaries. *Brain Struct. Funct.* 221 39–58. 10.1007/s00429-014-0891-9 25244943

[B35] Wang D. Guo Y. Li H. Li J. Ran M. Guo J. (2020). Selective optogenetic activation of orexinergic terminals in the basal forebrain and locus coeruleus promotes emergence from isoflurane anaesthesia in rats. *Br. J. Anaesth*. 126 279–292. 10.1016/j.bja.2020.09.037 33131759

[B36] Xu M. Chung S. Zhang S. Zhong P. Ma C. Chang W. C. (2015). Basal forebrain circuit for sleep-wake control. *Nat. Neurosci.* 18 1641–1647. 10.1038/nn.4143 26457552 PMC5776144

[B37] Xu W. Wang L. Yuan X. S. Wang T. X. Li W. X. Qu W. M. (2020). Sevoflurane depresses neurons in the medial parabrachial nucleus by potentiating postsynaptic GABA(A) receptors and background potassium channels. *Neuropharmacology* 181:108249. 10.1016/j.neuropharm.2020.108249 32931816

[B38] Yang S. R. Hu Z. Z. Luo Y. J. Zhao Y. N. Sun H. X. Yin D. (2018). The rostromedial tegmental nucleus is essential for non-rapid eye movement sleep. *PLoS Biol.* 16:e2002909. 10.1371/journal.pbio.2002909 29652889 PMC5919677

[B39] Zhang B. Gao Y. Li Y. Yang J. Zhao H. (2016). Sleep deprivation influences circadian gene expression in the lateral habenula. *Behav. Neurol.* 2016:7919534. 27413249 10.1155/2016/7919534 PMC4930817

[B40] Zhang Y. Fu B. Liu C. Yu S. Luo T. Zhang L. (2019). Activation of noradrenergic terminals in the reticular thalamus delays arousal from propofol anesthesia in mice. *FASEB J.* 33 7252–7260. 10.1096/fj.201802164rr 30860868

[B41] Zhao H. Rusak B. (2005). Circadian firing-rate rhythms and light responses of rat habenular nucleus neurons in vivo and in vitro. *Neuroscience* 132 519–528. 10.1016/j.neuroscience.2005.01.012 15802202

[B42] Zhong H. Tong L. Gu N. Gao F. Lu Y. Xie R. G. (2017). Endocannabinoid signaling in hypothalamic circuits regulates arousal from general anesthesia in mice. *J. Clin. Invest.* 127 2295–2309. 10.1172/jci91038 28463228 PMC5451249

