# Supplementary figures and images for: Lateral Habenula Glutamatergic Neurons Modulate Isoflurane Anesthesia in Mice
Source: Front Mol Neurosci. 2021 Mar 4;14:628996. doi: 10.3389/fnmol.2021.628996 (PMC7969819; doi:10.3389/fnmol.2021.628996)

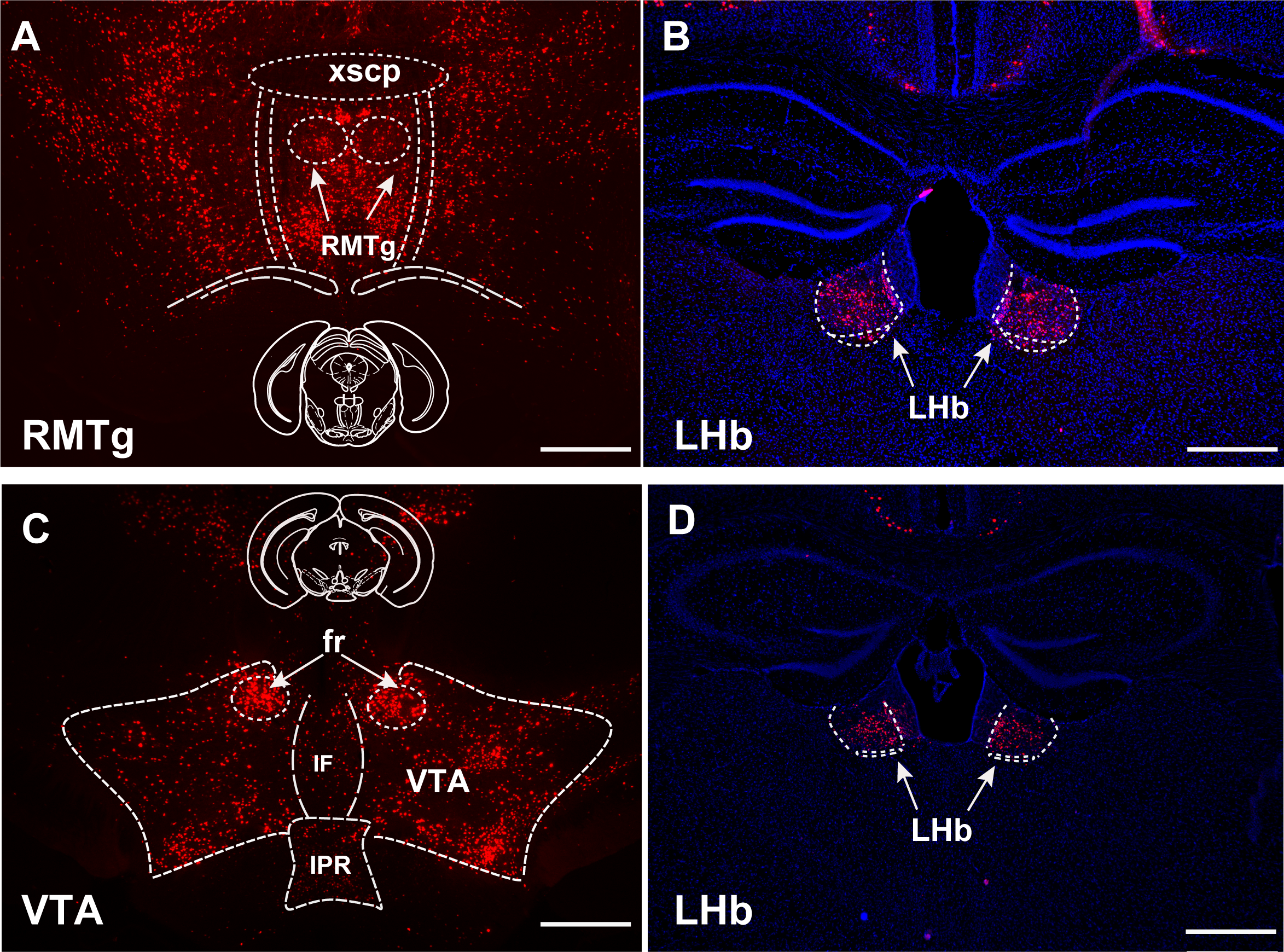

Supplement: Supplementary Figure 2 — (A) Representative injection of rAAV-retro-hSyn-CRE-mCherry-WPRE-hGH in RMTg. (B) A representative image of the LHb-projecting RMTg neurons. (C) Representative injection of rAAV-retro-hSyn-CRE-mCherry-WPRE-hGH in VTA. (D) A representative image of the LHb-projecting VTA neurons (scale bars = 400 μm). [file Image_2.TIF]

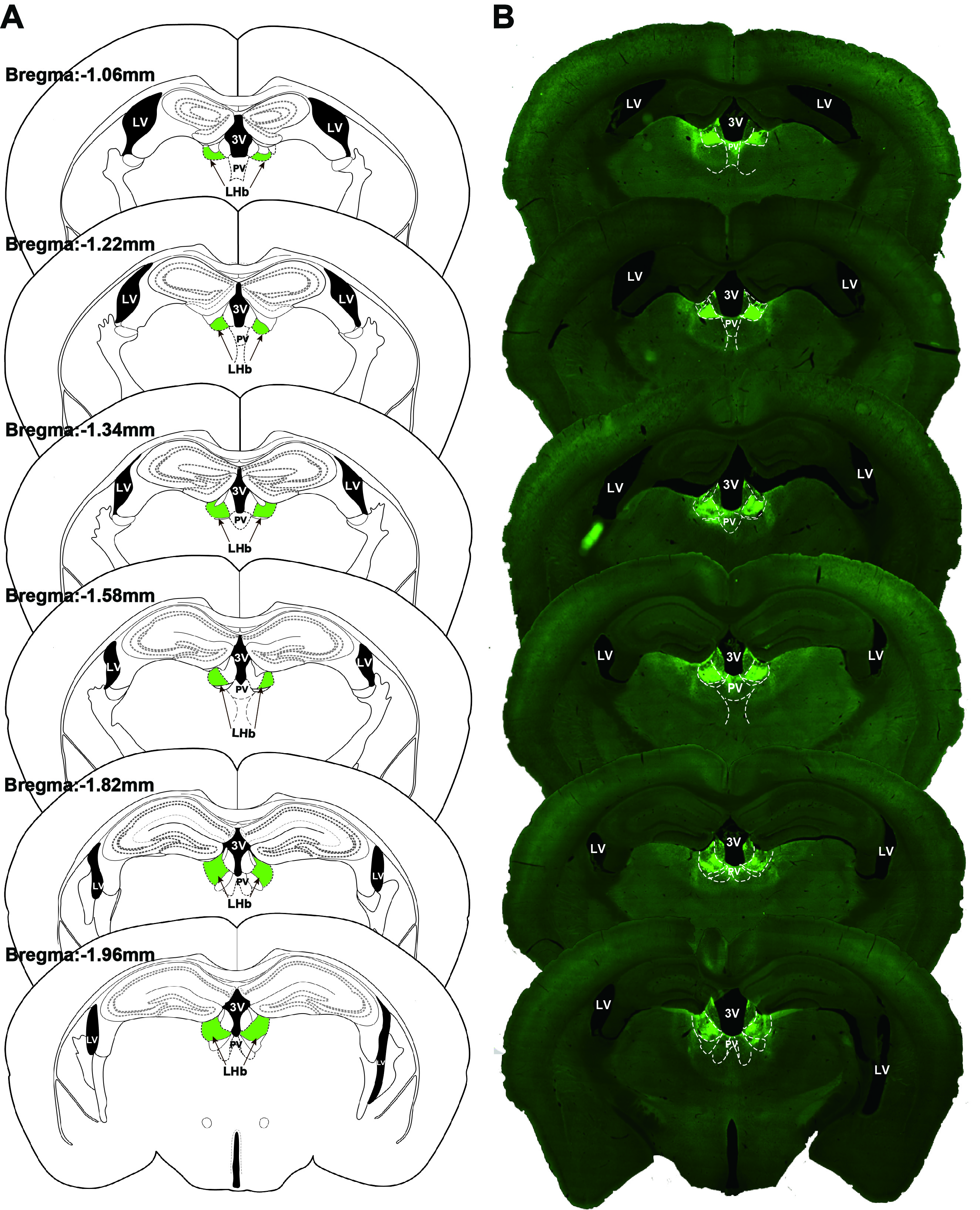

Supplement: Supplementary Figure 3 — Injection sites were localized to LHb. Virus-transfection sites from representative photomicrograph of a virus-stained coronal section throughout the rostral-caudal axis (A) LHb are represented by green on coronal schematics modified from the atlas of Franklin and Paxinos between bregma −1.06 mm and bregma −1.94 mm in mouse (Paxinos and Franklin, 2013). (B) Corresponding schematic of the virus transfections sites in LHb. [file Image_3.JPEG]
